# Effects of Pre-Operative HbA1c on Outcomes and the Rate of Clinical Improvement Following Anterior Cervical Discectomy and Fusion

**DOI:** 10.3390/jcm14134589

**Published:** 2025-06-28

**Authors:** Ara Khoylyan, Noah Coleman, Matthew Parry, Alex Tang, Tan Chen

**Affiliations:** 1 Geisinger Commonwealth School of Medicine, Scranton, PA 18509, USA; akhoylyan@som.geisinger.edu (A.K.);; 2 Geisinger Northeast Orthopaedic Surgery Residency, Wilkes-Barre, PA 18711, USAatang1@geisinger.edu (A.T.); 3 Department of Orthopaedic Surgery, Geisinger Medical Center, Danville, PA 17822, USA

**Keywords:** anterior cervical diskectomy and fusion, ACDF, diabetes mellitus, complications, outcomes, neck disability index, spine surgery

## Abstract

Retrospective Cohort Study. **Objectives:** The objectives of this study are to (1) compare post-operative patient-reported outcome measures (PROMs) between non-diabetic (non-DM) and diabetic (DM) patients undergoing Anterior Cervical Discectomy and Fusion (ADCF), (2) characterize the clinical trajectory, and (3) compare the rate of post-operative complications. **Methods:** A total of 261 non-DM and 52 DM patients were included. Patient demographics, Neck Disability Index (NDI) and Patient-Recorded Outcomes Measurement Information System (PROMIS) scores were collected up to one year after operation. Maximum medical improvement (MMI) was defined as the timepoint where more than 90% of the cohort achieved a minimal clinically important difference (MCID) in survey scores. Post-operative complications were collected. Descriptive and inferential statistics were performed. **Results:** Non-DM patients achieve MMI significantly more quickly than DM patients (non-DM: 6 months; DM: 1 year, *p* < 0.010). No difference in ∆NDI (non-DM: 24.9; DM: 23.0; *p* = 0.824) or ∆PROMIS-Physical Function (non-DM: 7.1; DM: 9.1; *p* = 0.373) was found between the two cohorts. In diabetic patients undergoing single-level fusion ACDF, a pre-operative HbA1c of ≥7.3% demonstrates 100% sensitivity and 25% specificity in detecting failure to achieve 1-year PROMIS MCID (AUC = 0.833, *p* = 0.009). There was no association between diabetic status and post-operative complication rate. **Conclusions:** Diabetic patients may demonstrate a slower rate of achieving maximum medical improvement despite equal subjective and clinical outcomes. Pre-operative HbA1c ≥ 7.3% demonstrates a significant correlation with worse subjective outcomes following single-level ACDF.

## 1. Introduction

Anterior Cervical Discectomy and Fusion (ACDF) is one of the most frequently performed spine operations in the United States, with recent estimates exceeding 150,000 procedures annually and aggregate inpatient costs surpassing USD 4 billion [1,2,3,4]. Common indications include disk herniation, spinal stenosis, spondylolisthesis, and scoliosis [5]. There are risks and complications associated with this procedure that are well known; typical complications include infection, hematoma, hardware failure, nerve damage, radicular pain, and dysphagia [6]. Despite its widespread success in alleviating radiculomyelopathic symptoms, a meaningful subset of patients experiences persistent neck-related disability or an unexpectedly protracted recovery—outcomes that threaten value-based care benchmarks and patient satisfaction. Consequently, optimizing modifiable risk factors before surgery has become a principal strategy in contemporary spine practice. Pre-operative health status is an important factor when considering optimal surgical planning to mitigate peri- and post-operative complications and improve recovery. Pre-operative comorbidities such as obesity, hypertension, cardiac dysfunction, lung disease, vascular abnormalities, and diabetes mellitus (DM) can greatly influence post-operative courses and complication rates [7,8]. DM has a progressively increasing prevalence in the United States, and there is a multitude of research demonstrating its effect on outcomes following spine fusion surgery [9,10].

Poorly controlled DM has been found to be associated with increased cardiac and vascular complications, respiratory distress, hematomas, transfusion rates, and mortality following spinal fusion [11,12,13]. Chronic hyperglycemia provokes micro- and macro-vascular alterations, impairs osteoblast activity, and attenuates immune competence, collectively jeopardizing spinal fusion biology and wound healing. In lumbar fusion cohorts, elevated pre-operative hemoglobin A1c (HbA1c) correlates with longer length of stay, hardware failure, and inferior patient-reported outcome measures (PROMs). Given this, prior studies have also implemented measurement of the minimal clinically important difference (MCID) of pre- and post-operative PROMs to gauge optimal pre-operative HbA1c levels for predicting post-ACDF recovery [14]. In contrast, there is also literature suggesting that there are no differences between the diabetic and non-diabetic surgical courses. Cervical-specific data, for example, are fragmented: some report higher dysphagia or infection rates among diabetic patients, whereas others fail to demonstrate clinically relevant differences [15]. Continuing to seek a better understanding of the post-operative clinical course of DM patients following ACDF surgery can lead to improvements in management of care, further limiting unwanted outcomes that culminate in increased medical interventions and costs.

Further investigation would be helpful in solidifying understanding about the impact of DM on the post-operative course following ACDF surgery, namely with reference to the rate of recovery. Maximum medical improvement (MMI) is a measurement that can evaluate the rate of recovery by measuring the time it takes for a patient group to reach peak improvement based on PROM MCID achievement rate [16,17,18]. The MMI following ACDF has been found to be at the 6-month post-operative timepoint based on NDI scores [17]. To the authors’ knowledge, no studies have distinguished MMI between diabetic and non-diabetic patients. This study seeks to (1) determine whether there is a difference in MMI based on diabetic status, and (2) further elucidate the impact of pre-operative HbA1c on post-operative outcomes including PROM scores and complications.

## 2. Methodology

Before any data was collected, an exemption from the local institutional review board was granted. A retrospective analysis assessed patients who had an elective single-level ACDF procedure between October 2019 and July 2023. Patients were initially identified using Current Procedural Terminology (CPT) codes for ACDF (22551). Patients at least 18 years of age who had a single or multi-level ACDF procedure for degenerative pathology with viable pre-operative HbA1c levels within two weeks and completed pre-operative and post-operative PROM surveys met the inclusion criteria. We excluded patients who had revisions at the level of the procedure or corpectomy procedure. Data was collected through manual chart review and included demographic factors including age, body mass index (BMI), gender, and surgical data.

### 2.1. Patient-Reported Outcome Measures (PROMs)

The Neck Disability Index (NDI) and Patient-Reported Outcomes Measurement Information System (PROMIS) Global Health, Physical Health, and Mental Health scores were obtained within one week pre-operatively and again post-operatively at six weeks, twelve weeks, six months, and one year. The minimum clinically important difference (MCID) was defined based on the previous literature in two ways: (1) an eight-point improvement in the NDI and PROMIS scores in comparison to the baseline pre-operative score defined as “MCID” and (2) a 30% improvement from the baseline pre-operative score defined as “MCID30” [14,19,20]. Maximum medical improvement was established as the timepoint at which 90% of the total population met MCID based on prior studies [17].

### 2.2. Statistical Analysis

Patients were divided into non-diabetic and diabetic cohorts based on pre-operative HbA1c ≥ 6.5% obtained within two weeks prior to surgery. Demographic data, follow-up time, surgical data (length of stay, blood loss, complications, number of levels fused), and PROM scores were compared between the two groups using Student’s *t*-test for continuous data and chi-squared for categorical data. Multivariate and univariate logistic regression analysis was completed between the pre-operative HbA1c levels or diabetic status as the independent variables and PROM scores and differences in surgical outcomes as the dependent variables to determine the odds ratio. Covariates in this analysis included age, gender, BMI, and number of levels fused. A receiver operator characteristic (ROC) analysis was performed to determine the area under the curve (AUC) and determine clinically sensitive and specific thresholds using pre-operative HbA1c as a predictor for post-operative outcomes. A ROC curve model quality > 0.5 was determined to be acceptable. PROMIS and NDI scores were compared between the DM and non-DM groups using a Student’s t-test. Finally, pre-operative diabetic status was compared with post-operative PROMIS and NDI scores using a Pearson’s correlation coefficient calculation. *p*-values ≤ 0.05 were determined to be statistically significant. All statistical calculations were completed using SPSS v25 (IBM Corporation, Armonk, NY, USA).

## 3. Results

**Figure 1:**
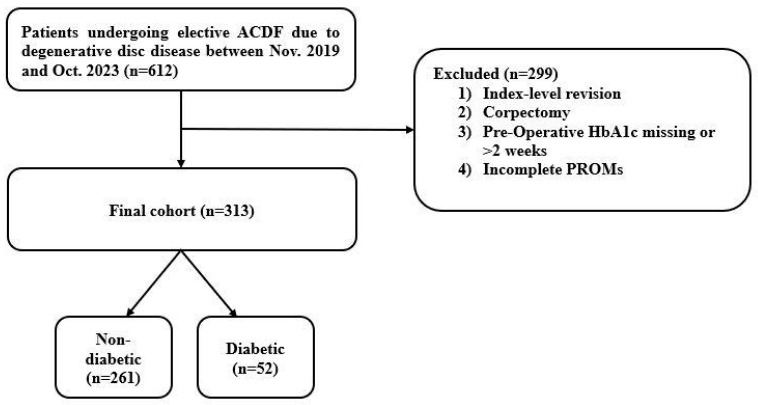
Determination of patient population based on eligibility criteria.

**Figure 2:**
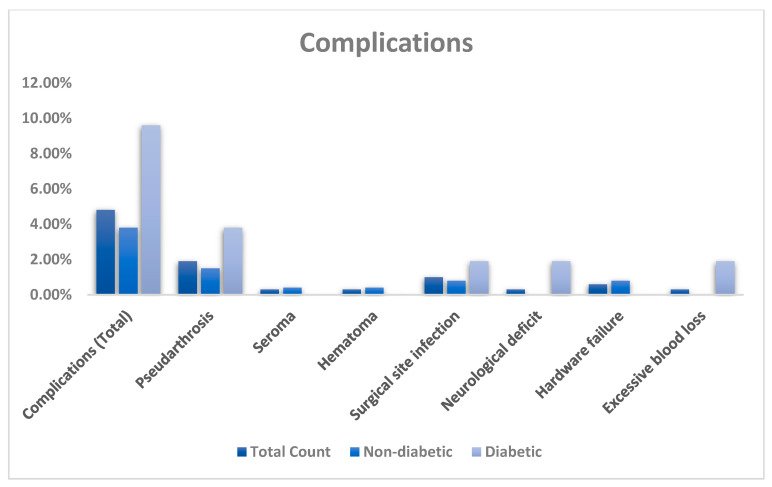
Comparison of rate of complications between diabetic and non-diabetic patients.

Of 612 patients identified, a total of 313 patients met the inclusion criteria and were included in this study (Figure 1). The population had a mean age of 55.7 ± 10.8 years and mean BMI of 31.1 ± 7.2 kg/m^2^. The distribution was 56% female and 44% male. The mean pre-operative HbA1c was 5.9 ± 0.8. There was a mean follow-up time of 20.9 ± 12.7 months and post-operative length of stay of 1.9 ± 0.6 days. Eleven (4%) patients required return to the operating room (O.R.). There were 108 (34%) single-level fusions and 205 (66%) multi-level fusions, with a mean number of levels fused of 1.9 ± 0.8 (Table 1). The complication rate in the total population was 15/313 (4.8%). The most common complication was pseudarthrosis (1.9%) (Table 2, Figure 2).

A total of 261 (83%) patients were categorized as non-DM and 52 (17%) as DM. The DM cohort was older (non-DM: 54.8 years; DM: 60.1 years, *p* < 0.001) and had higher BMI (non-DM: 30.3; DM: 35.4, *p* < 0.001). The mean post-operative length of stay was significantly longer in the DM cohort than non-DM (non-DM: 1.8; DM: 2.0, *p* < 0.047). There was no significant difference between the groups for mean number of fusion levels (non-DM: 1.9; DM: 1.8, *p* = 0.360), post-operative complications (non-DM: 4%; DM: 10%, *p* = 0.090), re-operation rate (non-DM: 2%; DM: 4%, *p* = 0.512); and follow-up time (non-DM: 20.5 months; DM: 22.5 months, *p* = 0.464) (Table 3).

**Figure 3:**
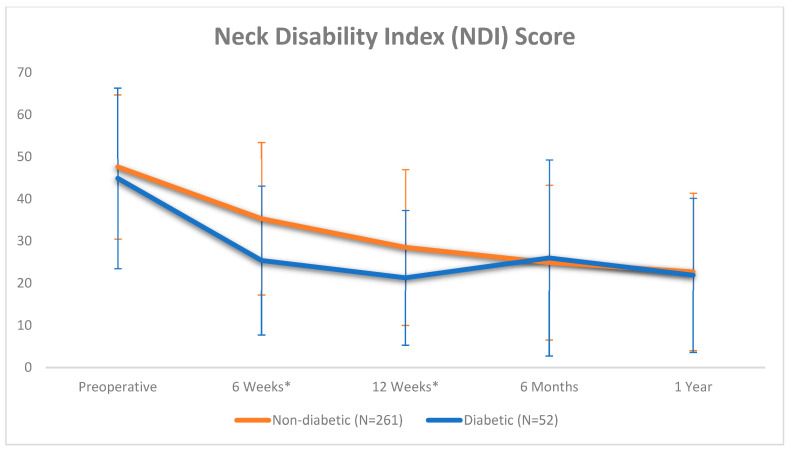
NDI score at each timepoint based on diabetic status. * Statistically significant difference in mean scores between diabetic and non-diabetic patients (*p* < 0.05).

The DM cohort had significantly lower mean NDI scores post-operatively at 6 weeks (non-DM: 35.3; DM: 25.4, *p* = 0.032) and 12 weeks (non-DM: 28.5; DM: 21.3, *p* = 0.011) (Table 4, Figure 3). There was no statistically significant score difference between the two cohorts at any other timepoint or on any other survey. The non-DM cohort had a significantly improved NDI score at each interval timepoint in comparison to the immediately prior timepoint (*p* < 0.05). The DM cohort only had significant improvement with NDI at the 6-week and 12-week timepoints in comparison to the immediate prior survey (Table 4).

**Figure 4:**
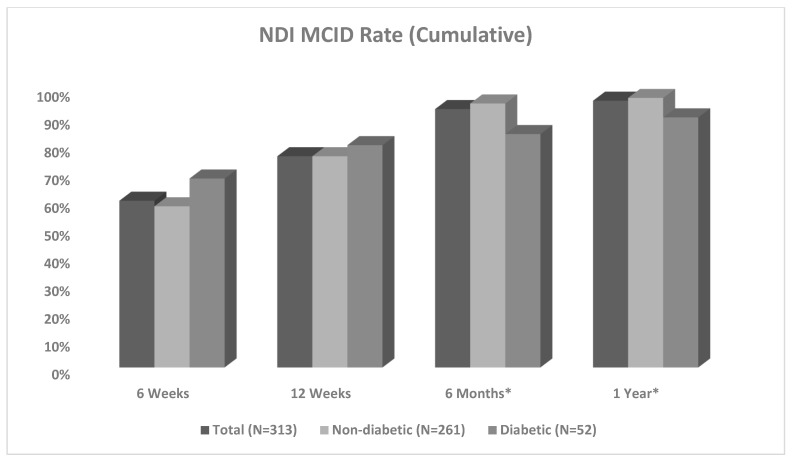
Comparison of cumulative NDI MCID achievement between the non-diabetic and diabetic cohorts. Note: Neck Disability Index (NDI); minimum clinically important difference (MCID) * Statistically significant difference in values between groups.

Non-DM patients demonstrated significantly greater cumulative NDI MCID achievement post-operatively by 6 months (non-DM: 95%, DM: 84%, *p* = 0.010) and 1 year (non-DM: 97%, DM: 90%, *p* = 0.049) (Figure 4). MMI based on NDI for the non-DM cohort was achieved at 6 months, while that for the DM cohort was achieved at 1 year (Table 5). DM patients were significantly less likely to achieve PROMIS Mental MCID at 1 year post-operatively (OR 0.176, CI 0.035–0.893, *p* = 0.036) (Table 6).

**Figure 5:**
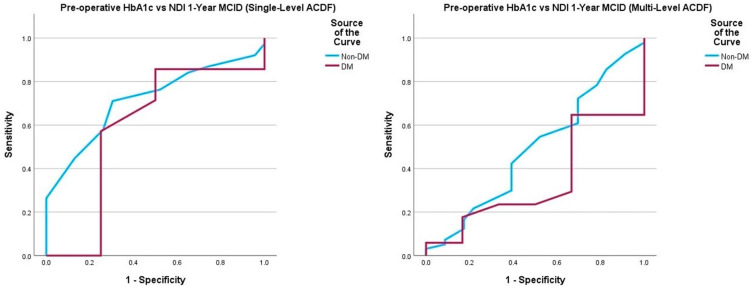
Comparison of ROC curve of pre-operative HbA1c and 1-year post-operative NDI MCID achievement between those undergoing single-level and multi-level ACDF. Note: Neck Disability Index (NDI); minimum clinically important difference (MCID).

**Figure 6:**
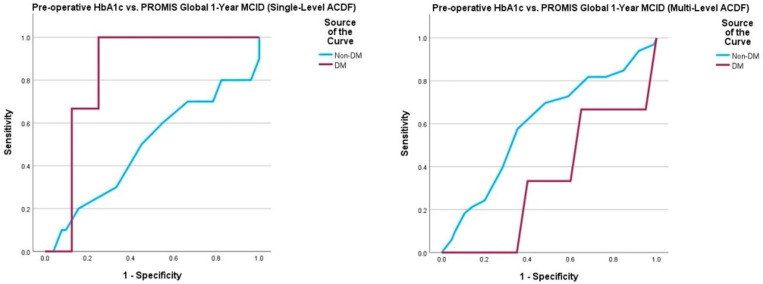
Comparison of ROC curve of pre-operative HbA1c and 1-year post-operative PROMIS Global MCID achievement between those undergoing single-level and multi-level ACDF. Note: Patient-Reported Outcomes Measurement Information System (PROMIS); minimum clinically important difference (MCID).

**Figure 7:**
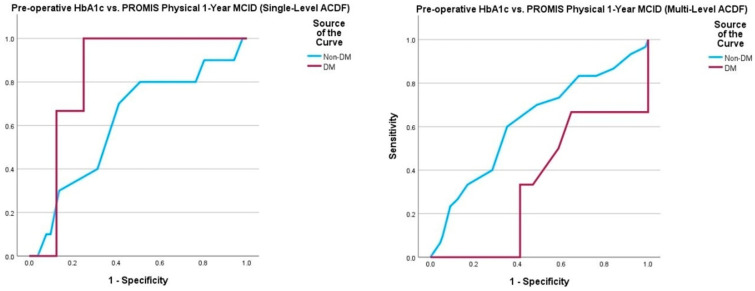
Comparison of ROC curve of pre-operative HbA1c and 1-year post-operative PROMIS Physical MCID achievement between those undergoing single-level and multi-level ACDF. Note: Patient-Reported Outcomes Measurement Information System (PROMIS); minimum clinically important difference (MCID).

**Figure 8:**
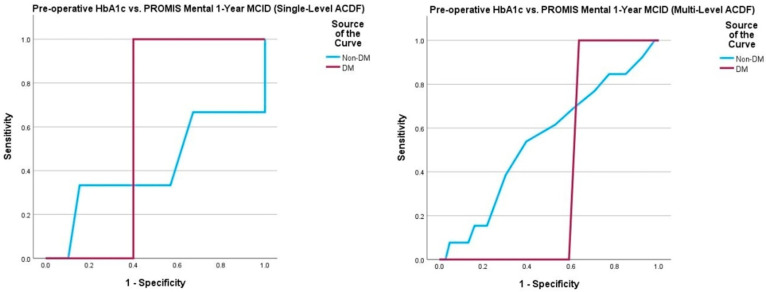
Comparison of ROC curve of pre-operative HbA1c and 1-year post-operative PROMIS Mental MCID achievement between those undergoing single-level and multi-level ACDF. Note: Patient-Reported Outcomes Measurement Information System (PROMIS); minimum clinically important difference (MCID).

Multivariate logistic regression yielded no statistically significant associations between pre-operative HbA1c and 1-year post-operative survey scores, peri- and post-operative complications, and need for return to the operating room (Table 7). In DM patients undergoing single-level ACDF, a pre-operative HbA1c value of 7.3% demonstrated 100% sensitivity and 25% specificity for detecting failure to achieve PROMIS Physical MCID30 (AUC = 0.833, *p* = 0.001). In non-DM patients undergoing single-level ACDF, a pre-operative HbA1c value of 5.5% demonstrated 71% sensitivity and 60% specificity for detecting failure to achieve NDI MCID30 (AUC = 0.716, *p* = 0.009). ROC curve analysis of the multi-level ACDF cohort did not yield any satisfactory thresholds (Table 8, Figure 5, Figure 6, Figure 7 and Figure 8).

## 4. Discussion

Diabetes is well known to negatively impact the peri- and post-operative course following surgical procedures including ACDF [10,21]. While outcomes in diabetic patients following ACDF surgery have been thoroughly explored, there is limited research on the impact of diabetic status and pre-operative HbA1c on post-operative rate of recovery. In our study, we evaluated the post-operative rate of recovery with MMI based on PROM data from our cohort of 313 patients and further investigated MCID to assess optimal pre-operative HbA1c thresholds, which have been cited in previous studies [14]. Our study expands on the prior literature by individually analyzing single- and multiple-level ACDF cohorts. Finally, we evaluated associations between pre-operative HbA1c and post-operative complications.

Although HbA1c is widely embraced as a surrogate of chronic glycemic exposure and is routinely checked by anesthesiologists to screen elective surgical candidates, its prognostic value in the cervical spine literature remains incompletely defined. Most available studies pool heterogeneous procedures—combining anterior and posterior approaches, single- and multi-level constructs, and even traumatic or oncologic indications—making it difficult to isolate the independent effect of pre-operative glycemic burden on recovery after ACDF specifically [14,22,23,24]. Furthermore, prior research has traditionally focused on binary end-points such as infection, readmission, or radiographic non-union which, although important, fail to capture the nuanced trajectory of symptom resolution now prioritized in value-based reimbursement models [25]. PROMs, particularly the NDI and PROMIS domains, offer a sensitive barometer of functional recovery, yet few datasets have linked these instruments with metabolic variables such as HbA1c [17]. Concurrently, the concept of maximum medical improvement has emerged as a pragmatic temporal benchmark that can guide surveillance schedules, shared decision-making, and bundled payment risk stratification [26]. Establishing whether glycemic status modifies the time required to reach MMI could therefore refine peri-operative counseling and allow more efficient allocation of rehabilitative resources. While established system guidelines or protocols exist for pre-operative glycemic management, they are not designed with longitudinal post-operative recovery in mind. Therefore, determining potential pre-operative screening HbA1c thresholds for prediction of post-operative recovery can be beneficial in further guiding pre-operative glycemic optimization, pharmacologic or otherwise. Given that contemporary diabetes management has evolved with the advent of glucagon-like peptide-1 receptor agonists and sodium–glucose cotransporter-2 inhibitors, it is beneficial to continuously update evidence that may guide modern pharmacologic control [27]. The present study addresses these gaps by analyzing a homogeneous ACDF cohort, integrating longitudinal PROM data with HbA1c values, and explicitly quantifying differences in MMI between diabetic and non-diabetic patients.

### 4.1. Rate of Recovery

Based on the results of their retrospective analysis of 69 patients undergoing ACDF, Patel et al. found the post-operative MMI for patients who underwent ACDF to be 6 months, and therefore recommended following up with PROMs for at least 6 months post-operatively [17]. This was in line with the results from our total population, in which MMI was also found to be at 6 months post-operatively. Sub-analysis of the 261 non-diabetic patients within this group also revealed MMI at the 6-month timepoint with an NDI MCID achievement rate of 95% of the population. However, investigating PROMs of the 52 diabetic patients revealed MMI to be at 1 year in this group, at which point 90% of the population had achieved MCID. These are novel findings that expand established literature by demonstrating that diabetic patients require significantly longer periods after ACDF surgery—perhaps up to 6 months longer—to reach peak recovery. This may serve as an important consideration in planning and managing expectations for the post-operative course. Measuring post-operative milestones through PROMs is an important part of surgical practice and can further guide post-operative care by streamlining workflows, supporting shared decision-making, and improving clinical care by reducing unnecessary surgeries and aligning treatments with patient goals [28]. Given the potentially slower rate of recovery, trending NDI in diabetic patients for longer periods than previously recommended for ACDF in general may augment the management of post-operative care, better identify potential complications, improve clinical outcomes, prompt multidisciplinary collaboration, and further establish trust in the patient–doctor relationship in this cohort [28,29]. However, while our findings may help guide post-operative considerations, future research with larger sample sizes and more balanced demographic characteristics (e.g., age, BMI) between diabetic and non-diabetic cohorts should be implemented before definitive conclusions about the rate of recovery can be reached. Additionally, it is important to note that to the authors’ knowledge, there are no well-defined criteria for the assessment of MMI following spine surgery. This serves as another important limitation to our findings, though these results may benefit future studies investigating ideal methods of measuring MMI in patients following ACDF.

### 4.2. Post-Operative Recovery Trend

Based on NDI scores, non-diabetic patients showed significant improvement between each interval timepoint while diabetic patients significantly improved from their immediate prior survey score only at the 6-week and 12-week post-operative timepoints, indicating a gap of six or more months following initial surveys in which their reported outcome scores were effectively unchanged. While the diabetic cohort did have significantly lower NDI scores at the 6-week and 12-week timepoints before equalizing, there were no significant differences in MCID achievement at these intervals and, as previously mentioned, non-diabetics reached MMI based on the target of 90% MCID more quickly (Figure 4). It should be noted that—given that a value of eight points or greater improvement is suggestive of MCID for NDI and PROMIS surveys—diabetic patients demonstrated a clinically significant improved mean NDI score at the 6-week timepoint in comparison to non-diabetics at the same interval. This was not the case for the 12-week timepoint. However, PROM scores at the 1-year timepoint were equal between the two cohorts on NDI and all three PROMIS surveys. Additionally, while these findings demonstrate that diabetics and non-diabetics achieve parity in subjective outcomes by the 1-year post-operative timepoint, recovery for non-diabetic patients may be steady and predictable, whereas recovery for those with diabetes may follow a more sporadic or unpredictable trend based on NDI (Figure 3). These findings may further strengthen the consideration for diabetic patients to be surveyed with PROMs for longer periods of time, given that early improvement may not necessarily represent long-term outcomes. Future studies may employ NDI PROMs past the 1-year mark to further elucidate the trajectory of recovery of diabetics after ACDF. Based on our current results, while consideration and discussion of these findings may be helpful in planning for the post-operative course and setting expectations for patients depending on their diabetic status, future research is necessary to elicit definitive adjustments in treatment management or recommendations, as previously mentioned.

### 4.3. Pre-Operative HbA1c and PROMs

Individual analysis of the diabetic cohort that underwent single-level ACDF demonstrated a pre-operative HbA1c threshold of 7.3% to have strong detection for failure to achieve 1-year post-operative PROMIS MCID based on Youden’s Index mining (sensitivity: 100%; specificity: 25%). Analysis of the multi-level ACDF cohort yielded poor results, suggesting that outcomes for those undergoing single-level ACDF may be more labile to pre-operative HbA1c values in comparison to multi-level ACDF. These findings may be due to the significantly poorer pre-operative status of those requiring multi-level ACDF. Diabetic patients who underwent multi-level ACDF in our study indeed had a significantly higher pre-operative NDI score than those undergoing single-level ACDF (44.5 vs. 50.1, *p* = 0.05), which may result in a greater capacity for surgical intervention to improve outcomes and minimize the impact of pre-operative HbA1c status. Given these findings in conjunction with our small sample size of patients with single-level ACDF in particular, future studies involving larger patient samples would be necessary and beneficial in further understanding differences in the impact of pre-operative HbA1c status based on the number of levels fused in ACDF. In comparison to our findings, current established pre-operative HbA1c thresholds for considering delay of elective surgery are ≥9.0% per the Australian Diabetes Society and ≥8.5% per the National Health Society and 2021 Centre for Perioperative Care guidelines [30,31,32]. While these results of the present study do not indicate the necessity for a delay of operation, they can establish more informed expectations for the post-operative course following single-level ACDF. Additionally, while our ROC analysis identified a pre-operative HbA1c threshold of 7.3% associated with delayed PROMIS recovery in diabetic patients undergoing single-level ACDF, the low specificity (25%) limits its clinical utility as a stand-alone decision-making tool. Rather, this threshold may serve as a reference for larger studies investigating this relationship and a potential signal for clinicians to initiate shared decision-making conversations and consider targeted pre-operative glycemic optimization.

In our total diabetic population of 52 patients, we also did not find a significant appropriate HbA1c threshold for assessing the risk of post-operative NDI MCID failure. We did however find a moderately satisfactory threshold of 5.5% for detecting failure to achieve 1-year post-operative NDI MCID following single-level ACDF in the non-DM cohort (Sens 70%, Spec 61%). A 2022 study by Roth et al. investigated 259 patients with diabetes undergoing anterior cervical fusion and demonstrated a pre-operative HbA1c threshold of 6.1% to be 93% sensitive and 61% specific in detecting failure to achieve NDI MCID at one year following anterior and posterior cervical laminectomy and fusion [14]. Roth et al. additionally demonstrated significantly lower odds of obtaining NDI MCID with increasing pre-operative HbA1c (OR 0.37, CI 0.22–0.62, *p* < 0.001). We found decreased odds in diabetics that were statistically significant only for the PROMIS Mental survey (OR 0.18, CI 0.04–0.89, *p* = 0.036). Differences in findings between studies can be attributed to several notable factors. The study by Roth et al. contained a larger database of diabetic patients than our own (259 vs. 52). Additionally, the previous study investigated outcomes in those undergoing both anterior and posterior cervical laminectomy and fusion, while we only investigated ACDF. Therefore, our findings do not discredit prior research due to these notable differences, and may prompt future investigations with larger cohorts to determine whether pre-operative HbA1c may have utility in predicting failure to meet NDI targets in all patients, including those who are not diabetic.

### 4.4. Post-Operative Complications

We did not find statistically significant relationships between pre-operative HbA1c or diabetic status and post-operative complications, including readmission for re-operation (Table 2, Figure 2). However, there was a trend towards significance with diabetics demonstrating a 2.5 times greater rate of experiencing a complication (non-DM: 4%; DM: 10%, *p* = 0.09). Roth et al. similarly found negative results, though reported a trend toward significantly greater odds of re-operation for patients with pre-operative HbA1c ≥ 6.8% (AUC = 0.61, 95%CI 0.52–0.69, *p* = 0.078) [14]. A meta-analysis including 22 studies by Tao et al. in 2023 indicated that patients with “preoperative HbA1c > 8.0% had increased risk(s) of postoperative complications (RR: 1.85, 95% CI: [1.48, 2.31], *p* < 0.01) and that patients with surgical site infection (SSI) had higher preoperative HbA1c (Mean Difference: 1.49%, 95% CI: [0.11, 2.88], *p* = 0.03).” [23]. Cancienne et al. published a retrospective study of 3341 ACDF patients in 2017 and demonstrated that those with a pre-operative HbA1c of ≥7.5 mg/dL were significantly more likely to have an SSI within 1 year of operation (AUC = 0.67; specificity, 68%; sensitivity, 46%; *p* = 0.022) [24]. There were only three reported SSIs in our study and there was no significant difference in rate between the two cohorts (non-DM: 0.8%; DM: 1.9%, *p* = 0.434). Of note, Tao et al. did not specifically evaluate cervical etiology, and the negative impact of pre-operative diabetic status on post-operative outcomes following lumbar surgery is well known [33,34,35]. Additionally, of our 313-patient population, only 10 patients had a pre-operative HbA1c > 8.0%. The combination of these factors likely accounts for differences in findings. Upon further review, in addition to the research of Cancienne et al., two other prior studies have evaluated the role of pre-operative HbA1c in determining complications specifically following ACDF. Walid et al. in 2010 and Liow et al. in 2018 both published retrospective studies consisting of 339 and 29 patients undergoing ACDF, respectively, and found no significant differences in post-operative length of stay between diabetic and non-diabetic patients [15,36]. In contrast, we found a slightly increased mean post-operative length of stay in diabetics (non-DM: 1.8 days; DM: 2.0 days, *p* = 0.047). These are important considerations, given that prior research has indeed shown diabetics to have up to almost USD 4000 more peri- or post-operative costs compared to non-diabetics due to factors including complications following spine surgery [21,36,37,38]. However, the current findings in the literature in terms of impacts on the complication rate following ACDF surgery seem to be mixed. Our findings seem to trend toward a significantly increased rate in diabetics, but the overall complication rate is quite low (15/313; 4.8%), so this study is likely not sufficiently powered to detect differences between the two cohorts. Further research with larger, multi-center population samples may be helpful to determine the predictive role of pre-operative HbA1c in this capacity.

### 4.5. Pathophysiologic Considerations

Hyperglycemia disrupts multiple phases of musculoskeletal healing. Experimental animal work and tissue-level assays demonstrate that sustained elevations in serum glucose impair neutrophil chemotaxis and phagocytosis, blunt macrophage polarization, and diminish fibroblast proliferation, collectively delaying the transition from inflammatory to reparative phases of healing [39]. Advanced glycation end-products accumulate in cancellous bone and compromise collagen cross-linking, resulting in inferior graft integration and slower osseous fusion [40]. Furthermore, diabetes-related microangiopathy attenuates regional blood flow, limiting oxygen tension at the fusion bed and contributing to the more protracted trajectory captured by our PROM data [41]. These mechanisms provide a biologic rationale for our clinical observation that DM patients require an additional time to reach MMI.

### 4.6. Future Directions

Several unanswered questions arise from our findings. First, it remains unclear whether aggressive pre-operative glycemic optimization—such as short-course basal-bolus insulin or sodium–glucose cotransporter-2 inhibitor therapy—can meaningfully shorten time to MMI in single-level ACDF. A prospective, randomized trial with serial continuous glucose monitoring and density-matching of fusion constructs would address this knowledge gap. Second, emerging biologics (e.g., recombinant human bone morphogenetic protein-2, teriparatide) may counteract the detrimental osseous effects of diabetes; stratified analyses could determine whether their benefit is more pronounced in patients exceeding the pre-operative HbA1c threshold. Finally, the external validity of our results should be tested in diverse practice environments and in patients undergoing motion-preserving procedures such as cervical disk arthroplasty with long-term surveillance to evaluate whether the early delay in recovery influences implant longevity, adjacent-segment disease, or late re-operation rates.

### 4.7. Limitations

Several limitations warrant consideration when interpreting our findings. First, the retrospective design is inherently vulnerable to selection and information bias. In conjunction with a relatively small sample size of diabetic patients, these two important limitations may deem the present study as better suited as a reference for future larger investigations than current definitive clinical decision-making. Although strict inclusion criteria and multivariable adjustment were applied, unmeasured confounders—such as duration of diabetes, insulin dependence, glycemic variability, socioeconomic status, body mass index, age, and adherence to rehabilitation—may still influence outcomes and limit the ability to draw definitive conclusions from these findings. Glycemic control was assessed solely by a single pre-operative HbA1c value; intra-operative glucose excursions and early post-operative hyperglycemia, both implicated in impaired wound healing, were not captured. All procedures were performed at a single rural academic center using relatively uniform graft and fixation constructs, limiting external validity to high-volume urban or community settings that employ different implants (e.g., stand-alone cages) or adjuncts (e.g., recombinant bone morphogenetic protein-2). Fourth, radiographic parameters—segmental lordosis, fusion status, and adjacent-segment degeneration—were not routinely obtained beyond one year; incorporating structural end-points could strengthen mechanistic inference. The modest sample size of the diabetic single-level subgroup may have reduced statistical power to detect rare complications such as surgical site infection. Additionally, limited references were available for the determination of MMI; while our chosen methodology was supported by a prior study, the findings are inherently limited due to the absence of established literature on the measurement of MMI following spine surgery. Finally, follow-up was capped at 12 months; longer surveillance is required to determine whether delayed MMI in diabetic patients influences late re-operation, implant longevity, or healthcare costs.

## 5. Conclusions

Both diabetic and non-diabetic patients may achieve similar long-term clinical benefits following single- and multi-level ACDF for degenerative pathology, with no apparent differences in PROM scores one year post-operatively. However, diabetic patients may take longer post-operatively to achieve maximum medical improvement. In diabetic patients undergoing single-level ACDF, a pre-operative HbA1c threshold of ≥7.3% is correlated with poorer post-operative recovery. However, given the noteworthy confounding elements present in this study, a causal relationship between diabetic status and post-ACDF recovery cannot be established, and caution should be taken in interpreting these findings for application in the clinical setting. These results should be more appropriately applied in prompting further investigation into peri-operative glycemic optimization. Given the observational nature of this study and its limitations, the results are hypothesis-generating and should be validated in larger, prospective cohorts.

## Figures and Tables

**Table 1:** Descriptive characteristics of total patient population.

Variable	Mean or Total Count N = 313
Age	55.7 ± 10.8
BMI	31.1 ± 7.2
Female Male	176 (56%) 137 (44%)
Pre-Operative HbA1c	5.9 ± 0.8
Diabetic Status Diabetic Non-Diabetic	52 (17%) 261 (83%)
Follow-Up Time (Months)	20.9 ± 12.7
Length of Stay (Days)	1.9 ± 0.6
Post-Operative Complications Yes No	15 (5%) 298 (95%)
Return to Operating Room Yes No	11 (4%) 302 (96%)
Fusion(s) Single Multiple	108 (34%) 205 (66%)
Number of Levels Fused 1 2 3 4	108 (34%) 134 (43%) 59 (19%) 13 (4%)
Mean Number of Levels Fused	1.9 ± 0.8

Note: body mass index (BMI).

**Table 2:** Distribution of peri- and post-operative complications based on diabetic status.

	Total Count N = 313	Non-Diabetic N = 261	Diabetic N = 52	*p*-Value
Complication (total)	15 (4.8%)	10 (3.8%)	5 (9.6%)	0.090
Pseudarthrosis	6 (1.9%)	4 (1.5%)	2 (3.8%)	0.267
Seroma	1 (0.3%)	1 (0.4%)	0	
Hematoma	1 (0.3%)	1 (0.4%)	0	
Surgical site infection	3 (1.0%)	2 (0.8%)	1 (1.9%)	0.434
Neurological deficit	1 (0.3%)	0	1 (1.9%)	
Hardware failure	2 (0.6%)	2 (0.8%)	0	
Excessive blood loss	1 (0.3%)	0	1 (1.9%)	

**Table 3:** Descriptive statistics comparing diabetic and non-diabetic patient cohorts.

	Non-Diabetic (A1c < 6.5) N = 261	Diabetic (A1c ≥ 6.5) N = 52	*p*-Value
Age	54.8 ± 10.6	60.1 ± 10.5	<0.001
BMI	30.3 ± 6.7	35.4 ± 7.9	<0.001
Sex Female Male	151 (57%) 112 (43%)	26 (50%) 26 (50%)	0.325
Follow-Up Time (Months)	20.5 ± 12.4	22.5 ± 14.2	0.464
Length of Stay (Days)	1.8 ± 0.6	2 ± 0.8	0.047
Post-Operative Complications Yes No	10 (4%) 251 (96%)	5 (10%) 47 (90%)	0.090
Return to Operating Room Yes No	6 (2%) 255 (98%)	2 (4%) 50 (96)	0.512
Fusion(s) Single Multiple	89 (34%) 174 (66%)	20 (38%) 32 (62%)	0.522
Number of Levels Fused 1 2 3 4	90 (34%) 109 (41%) 53 (20%) 11 (5%)	19 (37%) 25 (48%) 6 (12%) 2 (3%)	0.523
Mean Number of Levels Fused	1.9 ± 0.8	1.8 ± 0.9	0.360

Note: body mass index (BMI).

**Table 4:** Comparing post-operative survey scores at each timepoint between diabetic and non-diabetic patients.

PROM	Total N = 313	Non-Diabetic (A1c < 6.5) N = 261	Diabetic (A1c ≥ 6.5) N = 52	*p*-Value
NDI Pre-op 6 Weeks 12 Weeks 6 Months 1 Year	47.1 ± 17.9 33.5 ± 18.4 27.2 ± 18.2 25.1 ± 19.3 22.6 ± 18.6	47.6 ± 17.1 35.3 ± 18.1 * 28.5 ± 18.5 * 24.9 ± 18.4 * 22.7 ± 18.7 *	44.9 ± 21.4 25.4 ± 17.7 * 21.3 ± 16.0 26.0 ± 23.3 21.9 ± 18.3 *	0.312 0.032 0.011 0.752 0.824
PROMIS Global Pre-op 6 Weeks 12 Weeks 6 Months 1 Year	29.2 ± 6.7 33.4 ± 7.4 33.3 ± 7.7 33.5 ± 8.0 33.5 ± 8.3	29.2 ± 6.5 33.0 ± 7.2 * 33.3 ± 7.7 * 33.7 ± 7.8 33.4 ± 8.1	29.4 ± 7.4 35.0 ± 8.4 * 33.5 ± 7.7 32.3 ± 8.4 33.6 ± 9.3	0.636 0.433 0.366 0.358 0.373
PROMIS Physical Pre-op 6 Weeks 12 Weeks 6 Months 1 Year	38.1 ± 6.6 44.8 ± 7.9 45.1 ± 8.3 45.4 ± 8.9 45.5 ± 9.2	38.2 ± 6.5 44.5 ± 7.6 * 44.9 ± 8.2 * 45.7 ± 8.6 45.3 ± 8.9	37.7 ± 7.1 46.1 ± 9.1 * 46.1 ± 8.5 * 44.2 ± 10.1 * 46.8 ± 10.5	0.636 0.433 0.366 0.358 0.373
PROMIS Mental Pre-op 6 Weeks 12 Weeks 6 Months 1 Year	44.0 ± 8.4 47.0 ± 8.6 46.8 ± 9.4 46.8 ± 9.6 46.8 ± 10.0	43.8 ± 8.1 46.5 ± 8.3 * 46.7 ± 9.4 47.0 ± 9.6 46.8 ± 9.9	44.8 ± 9.9 49.4 ± 9.7 * 47.3 ± 9.7 46.1 ± 9.7 47.0 ± 11.1	0.406 0.175 0.676 0.599 0.911

Note: Neck Disability Index (NDI); Patient-Reported Outcomes Measurement Information System (PROMIS). * Significant difference in mean score (*p* < 0.05) in comparison to survey at immediate prior timepoint.

**Table 5:** Determining differences in MMI based on cumulative proportion of patient cohort that met MCID at each timepoint.

Timepoint MCID Met	Total N = 313	Non-Diabetic (A1c < 6.5) N = 261	Diabetic (A1c ≥ 6.5) N = 52	*p*-Value
NDI 6 Weeks 12 Weeks 6 Months 1 Year	63/105 (60%) 230/301 (76%) 284/306 (93%) 299/313 (96%)	50/86 (58%) 190/251 (76%) 241/255 (95%) 252/261 (97%)	13/19 (68%) 40/50 (80%) 43/51 (84%) 47/52 (90%)	0.408 0.513 0.010 0.049
PROMIS Global 6 Weeks 12 Weeks 6 Months 1 Year	16/105 (15%) 80/299 (26%) 96/304 (32%) 115/313 (37%)	13/86 (13%) 68/249 (37%) 81/253 (47%) 96/261 (49%)	3/19 (32%) 12/50 (48%) 15/51 (51%) 19/52 (62%)	0.941 0.629 0.715 0.958
PROMIS Physical 6 Weeks 12 Weeks 6 Months 1 Year	28/105 (27%) 115/299 (38%) 144/304 (47%) 160/313 (51%)	22/86 (13%) 91/249 (37%) 118/253 (47%) 128/261 (49%)	6/19 (32%) 24/50 (48%) 26/51 (51%) 32/52 (62%)	0.592 0.128 0.571 0.105
PROMIS Mental 6 Weeks 12 Weeks 6 Months 1 Year	11/105 (10%) 61/299 (20%) 76/304 (25%) 94/313 (30%)	8/86 (9%) 51/249 (20%) 66/254 (26%) 83/261 (32%)	3/19 (16%) 10/50 (20%) 10/51 (20%) 11/52 (21%)	0.403 0.938 0.337 0.126

Minimal clinically important difference (MCID); Neck Disability Index (NDI); Patient-Reported Outcomes Measurement Information System (PROMIS).

**Table 6:** Relationship between diabetic status and 1-year post-operative MCID achievement. Covariates include age, BMI, sex, number of levels fused, and complications.

Diabetic Status				
1-Year Survey	Multivariate OR	*p*-Value	Univariate OR	*p*-Value
NDI MCID MCID30	2.585 0.716	0.346 0.606	1.733 0.818	0.391 0.626
PROMIS Global MCID MCID30	1.153 1.075	0.804 0.904	0.708 0.678	0.449 0.420
PROMIS Physical MCID MCID30	3.046 2.328	0.076 0.150	1.594 1.251	0.215 0.601
PROMIS Mental MCID MCID30	0.176 1.127	0.036 0.897	0.224 0.641	0.047 0.565

Note: minimal clinically important difference (MCID); Neck Disability Index (NDI); Patient-Reported Outcomes Measurement Information System (PROMIS).

**Table 7:** Logistic regression evaluating association between pre-operative HbA1c and MCID achievement on each survey at 1 year, peri- or post-operative complications, return to O.R., surgery type (single- vs. multi-level fusion), and post-operative length of stay (days). Covariates include age, BMI, and sex.

Pre-Operative HbA1c				
	DM (HbA1c ≥ 6.5%) N = 52		Non-DM (HbA1c < 6.5%) N = 261	
1-Year Survey	Multivariate OR	*p*-Value	Multivariate OR	*p*-Value
NDI MCID MCID30	3.814 0.884	0.357 0.806	0.715 1.150	0.661 0.825
PROMIS Global MCID MCID30	0.687 0.777	0.501 0.645	2.859 2.829	0.075 0.085
PROMIS Physical MCID MCID30	0.441 0.347	0.180 0.155	1.977 4.866	0.210 0.015
PROMIS Mental MCID MCID30	0.691 0.691	0.772 0.772	1.253 0.922	0.708 0.929
Complications	0.365	0.373	0.223	0.287
Return to OR	0.593	0.526	0.214	0.308

Note: minimal clinically important difference (MCID); Neck Disability Index (NDI); Patient-Reported Outcomes Measurement Information System (PROMIS); operating room (OR).

**Table 8:** ROC analysis evaluating predictive capability of pre-operative HbA1c in diabetic patients for failure to achieve minimum clinically important difference in survey score at 1-year post-operative timepoint distinguished by single- vs. multiple-level fusions.

	Single-Level Fusion					Multiple-Level Fusion				
MCID 30 (1-Year)	N	AUC	*p*-Value	Cutoff	Sens, Spec	N	AUC	*p*-Value	Cutoff	Sens, Spec
NDI (Non-DM)	57	0.716	0.009	5.05 5.45	0.92, 0.05 0.71, 0.60	174	0.490	0.889	5.15 5.45	0.88, 0.19 0.61, 0.38
NDI (DM)	20	0.589	0.664	6.80 7.30	0.86, 0.50 0.57, 0.75	32	0.343	0.205	5.15 5.45	0.88, 0.14 0.67, 0.24
PROMIS Overall (Non-DM)	57	0.489	0.920	3.90 5.55	1.00, 0.00 0.50, 0.65	174	0.598	0.096	5.05 5.65	0.94, 0.08 0.58, 0.65
PROMIS Overall (DM)	20	0.833	0.009	7.30 7.85	1.00, 0.25 0.67, 0.88	32	0.342	0.326	7.00 7.40	0.67, 0.35 0.33, 0.60
PROMIS Physical (Non-DM)	57	0.619	0.098	5.15 5.55	0.90, 0.20 0.70, 0.59	174	0.621	0.049	5.15 5.95	0.89, 0.14 0.34, 0.84
PROMIS Physical (DM)	20	0.833	0.001	7.30 7.85	1.00, 0.25 0.67, 0.88	32	0.338	0.206	5.50 7.00	1.00, 0.00 0.67, 0.34
PROMIS Mental (Non-DM)	57	0.417	0.690	5.15 5.45	0.85, 0.15 0.70, 0.49	174	0.544	0.596	5.15 5.45	0.82, 0.75 0.79, 0.44
PROMIS Mental (DM)	20	0.600	0.519	7.30 -	1.00, 0.40 -	32	0.386	0.277	7.00 -	1.00, 0.36 -

## Data Availability

The authors declare that all data used for analysis in this study will be made available upon appropriate request.
